# Supplementary material for: Tolerance of Plasmodium falciparum mefloquine-resistant clinical isolates to mefloquine-piperaquine with implications for triple artemisinin-based combination therapies
Source: Nat Commun. 2025 Nov 27;16:10634. doi: 10.1038/s41467-025-65629-8 (PMC12660809; doi:10.1038/s41467-025-65629-8)
Supplement: Supplementary file 1 — Supplementary Information [file 41467_2025_65629_MOESM1_ESM.pdf]

Tolerance of *Plasmodium falciparum* mefloquine-resistant clinical isolates to mefloquine-piperaquine with implications for triple artemisinin-based combination therapies.

Camille Roesch[a,Δ], Anna Cosson[b], Melissa Mairet Khedim[a], Nimol Khim[a], Nimol Kloeung[a], Sopheakvatey Ke[a], Sreynet Srun[a], Rotha Eam[a], Chanra Khean[a], Chanvong Kul[a], Lucie Adoux[c], Jean Popovici[a,d], Rithea Leang[e], Pascal Ringwald[f], Frédéric Ariey[g,h], Romain Coppée[b,i,j], Benoit Witkowski[a,Δ,ɫ,*]

[a]Malaria Unit, Pasteur Institute of Cambodia, Phnom Penh, Cambodia

[b]Laboratoire de Parasitologie-Mycologie, UR ESCAPE, Université de Rouen Normandie, Rouen, France.

[c]GENOM'IC, Université Paris Cité, CNRS, INSERM, Institut Cochin, F-75014 Paris, France.

[d]Infectious Disease Epidemiology and Analytics G5, Department of Global Health, Institut Pasteur, Université Paris Cité, INSERM U1347, Paris, France.

[e]National Center for Parasitology, Entomology, and Malaria Control, Ministry of Health, Phnom Penh, 120801, Cambodia.

[f]Mekong Malaria Elimination Programme, WHO, Phnom Penh, Cambodia.

[g]INSERM U1344, MERIT IRD, Université Paris Cité, Paris, France.

[h]Service de Parasitologie-Mycologie, Hôpital Cochin, Paris, France.

[i]Centre National de Référence du Paludisme, Laboratoire de Parasitologie-Mycologie, Hôpital Bichat-Claude Bernard, Paris, France.

[j]Centre National de Référence Cryptosporidioses, microsporidies et autres protozooses digestives, Centre Hospitalier Universitaire de Rouen, Rouen, France.

[Δ]Present affiliation : Genetic and Biology of Plasmodium Unit, Institut Pasteur de Madagascar, Antananarivo, Madagascar & PV-ESMEE Pasteur International Unit, Institut Pasteur de

Madagascar, Antananarivo, Madagascar ; Pasteur Institute, Paris, France ; Pasteur Institute of Cambodia, Phnom Penh, Cambodia.

ǂPresent affiliation : Infectious Disease Epidemiology and Analytics G5, Department of Global Health, Institut Pasteur, Université Paris Cité, INSERM U1347, Paris, France

*Address correspondence to Benoit Witkowski bwitkowski@pasteur.mg

Supplementary Figure 1. Summary of pressure experiment. Source data are provided as a Source Data file.

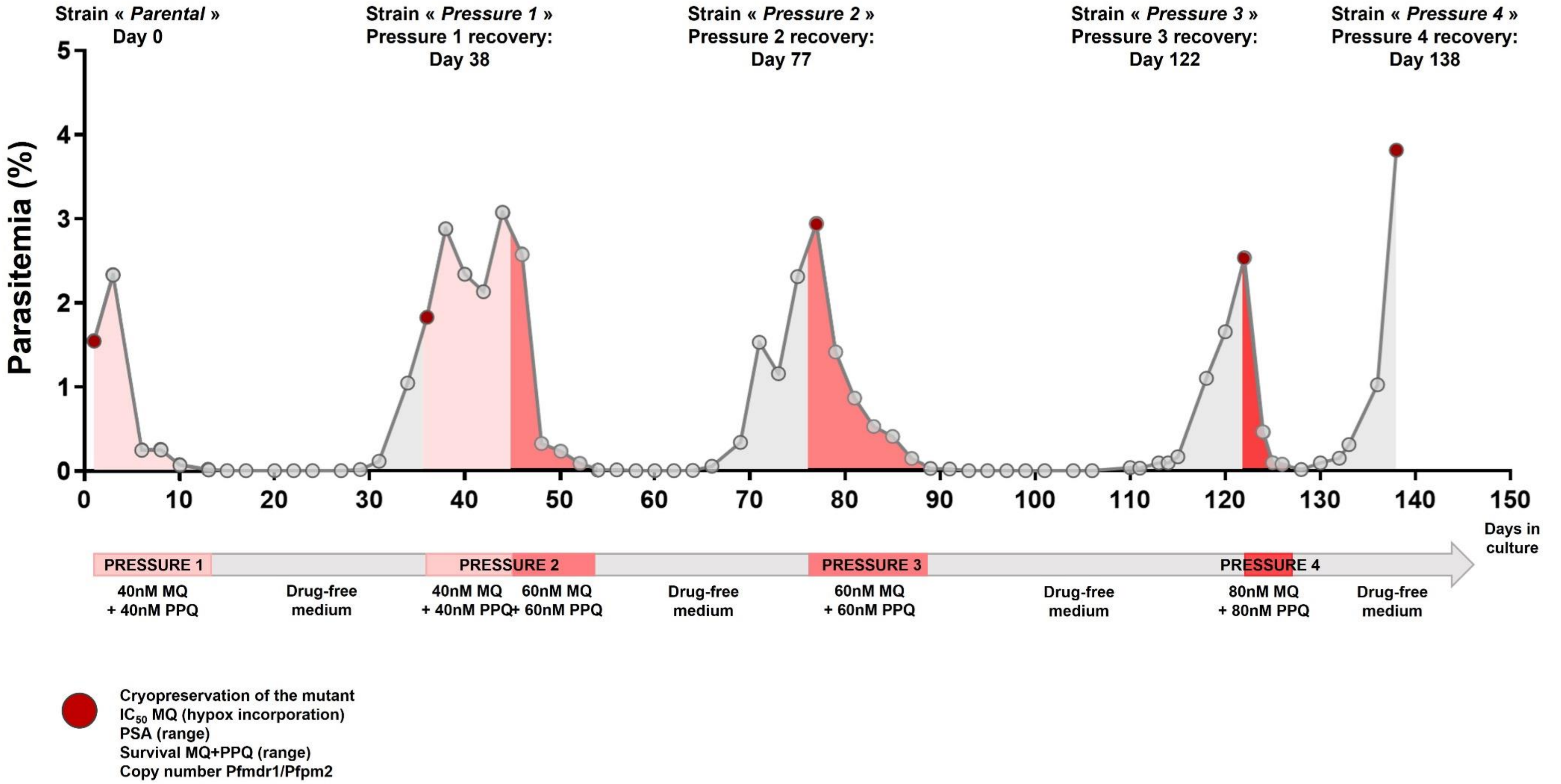

Supplementary Figure 2. Evolution of the *in vitro* susceptibility of the parent strain and the selected strain after continuous pressure of MQ+PPQ, measured by conventional methods. Mean and standard deviation are represented on the graphs and each dot represents one independent biological replicate. P-values are based on two-sided tests.

A. MQ susceptibility evolution. The susceptibility to MQ was measured using incorporation of $[^3H]$-hypoxanthine and IC_{50s} were calculated (**** $p = 4.72 \times 10^{-6}$, unpaired *t*-test – Shapiro-Wilk test p-value = 0.7641 and 0.6358 respectively for *Parental* (n = 8, grey) and *Pressure 4* (n = 4, pink)). **B.** PPQ susceptibility evolution. The susceptibility to PPQ was determined using PSA. A significant increase was observed between the two strains, but PSA remains below the threshold of resistance (** $p = 0.0068$, Mann-Whitney *U* test – Shapiro-Wilk test *p*-value = 0.0321 and 0.2987, respectively for *Parental* (n = 10, grey) and *Pressure 4* (n = 7, pink). Since only the *Parental* group passed the Shapiro-Wilk normality test and not the *Pressure 4*, we decided to use non-parametric test). Zeros shown as 0.001 for log scale purposes. Source data are provided as a Source Data file.

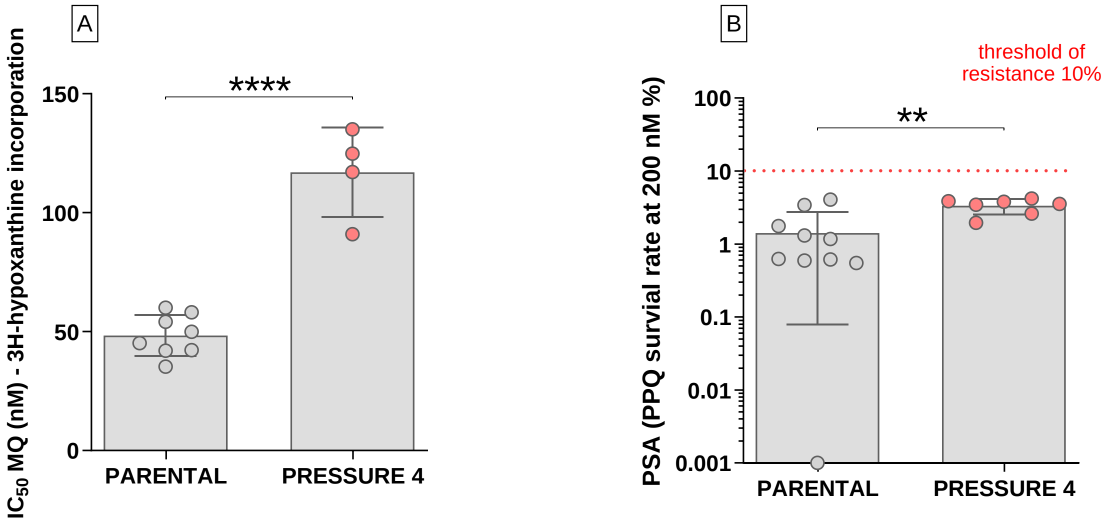

Supplementary Figure 3. Number of (A) SNPs and (B) indels detected between the two studied strains (*Parental* and *Pressure 4* strains). The plots report the number of genomic positions retained after each filtering step.

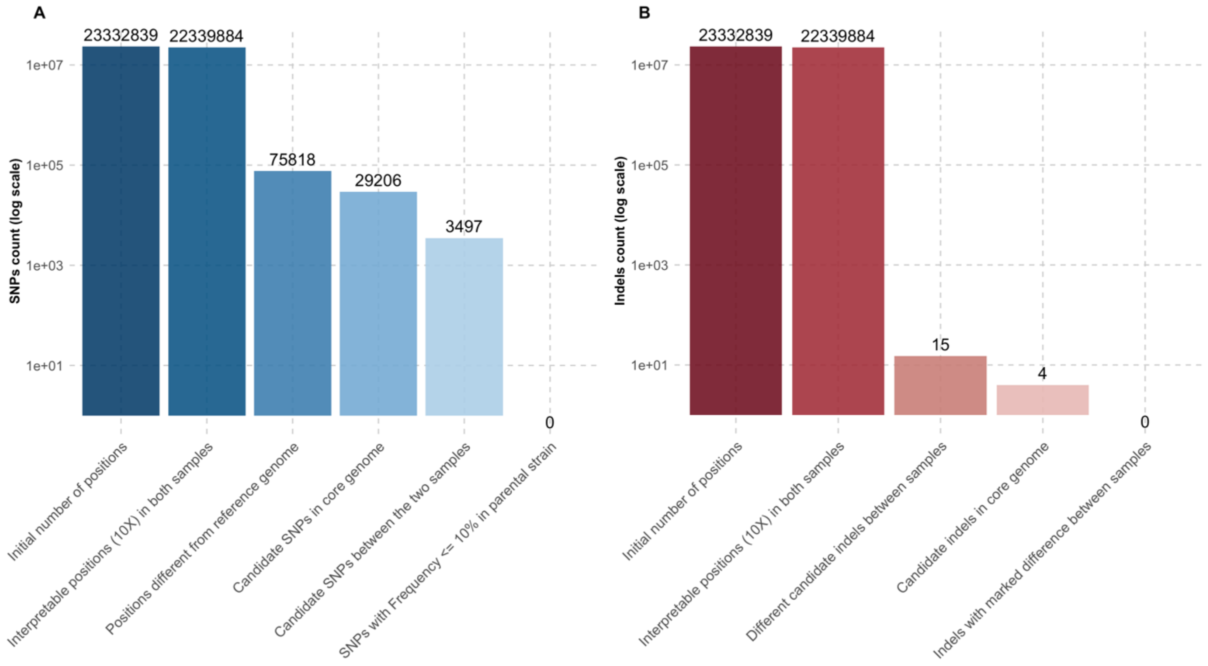

Supplementary Figure 4. Genomic characterization of amplified region on *Plasmodium falciparum* on chromosome 5 across laboratory and field isolates. Comparative genomic profiles are shown as vertical bars across the 800-980 kb region of chromosome 5. The Cambodian P4 line corresponds to the sample *Pressure 4* that was under MQ+PPQ pressure. The group labeled Cambodian, PPQ-R corresponds to four recently collected Cambodian isolates from 2019, all phenotypically resistant to PPQ as determined by PSA (range between 25.61% and 64.18%). These isolates represent the most recent PPQ-resistant parasites available from our own collection in Cambodia. DNA extraction and sequencing were performed using the same procedure as applied to the *Pressure 4* strain (see Materials and Methods). Importantly, no amplification events were detected in this genomic region for these PPQ-R isolates, contrasting with the *Pressure 4* strain. For comparison, data from Veiga *et al.* [1] (shown in pink) represent previously characterized Asian parasite lines (laboratory (FCB, FCR3, F32, and Dd2) and field isolates from Thailand), while the orange profile corresponds to a Dd2-derived line exposed to sustained PPQ pressure, as described by Eastman *et al.* [2] All annotated genes within this locus are shown, with their PlasmoDB identifiers indicated. The multidrug resistance gene 1 (*mdr1*, PF3D7_0523000) is specifically highlighted in red and boxed.

1. Veiga, M. I. *et al. pfmdr1* Amplification Is Related to Increased Plasmodium falciparum In Vitro Sensitivity to the Bisquinoline Piperaquine. *Antimicrob Agents Chemother* **56**, 3615–3619 (2012).
2. Eastman, R. T., Dharia, N. V., Winzeler, E. A. & Fidock, D. A. Piperaquine Resistance Is Associated with a Copy Number Variation on Chromosome 5 in Drug-Pressured *Plasmodium falciparum* Parasites. *Antimicrob Agents Chemother* **55**, 3908–3916 (2011).

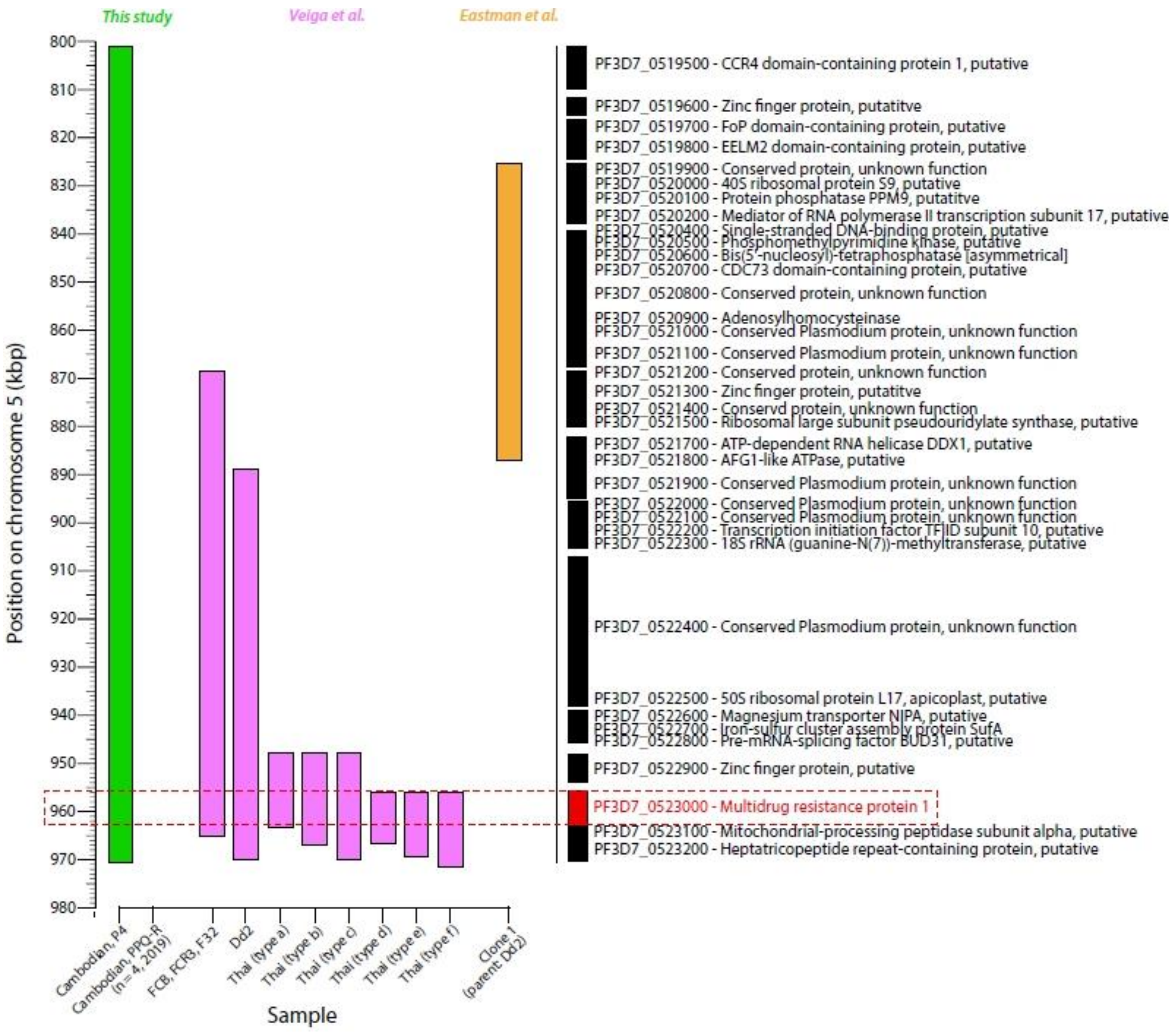
This study
Veiga et al.
Eastman et al.
Position on chromosome 5 (kbp)
800
810
820
830
840
850
860
870
880
890
900
910
920
930
940
950
960
970
980
Cambodian, P4
Cambodian, PPQ-R (n = 4, 2019)
FCB, FCR3, F32
Dd2
Thai (type a)
Thai (type b)
Thai (type c)
Thai (type d)
Thai (type e)
Thai (type f)
Clone 1 (parent: Dd2)
Sample
PF3D7_0519500 - CCR4 domain-containing protein 1, putative
PF3D7_0519600 - Zinc finger protein, putatitve
PF3D7_0519700 - FoP domain-containing protein, putative
PF3D7_0519800 - EELM2 domain-containing protein, putative
PF3D7_0519900 - Conserved protein, unknown function
PF3D7_0520000 - 40S ribosomal protein S9, putative
PF3D7_0520100 - Protein phosphatase PPM9, putatitve
PF3D7_0520200 - Mediator of RNA polymerase II transcription subunit 17, putative
PF3D7_0520400 - Single-stranded DNA-binding protein, putative
PF3D7_0520500 - Phosphomethylpyrimidine kinase, putative
PF3D7_0520600 - Bis(5'-nucleosyl)-tetraphosphatase [asymmetrical]
PF3D7_0520700 - CDC73 domain-containing protein, putative
PF3D7_0520800 - Conserved protein, unknown function
PF3D7_0520900 - Adenosylhomocysteinase
PF3D7_0521000 - Conserved Plasmodium protein, unknown function
PF3D7_0521100 - Conserved Plasmodium protein, unknown function
PF3D7_0521200 - Conserved protein, unknown function
PF3D7_0521300 - Zinc finger protein, putatitve
PF3D7_0521400 - Conservd protein, unknown function
PF3D7_0521500 - Ribosomal large subunit pseudouridylate synthase, putative
PF3D7_0521700 - ATP-dependent RNA helicase DDX1, putative
PF3D7_0521800 - AFG1-like ATPase, putative
PF3D7_0521900 - Conserved Plasmodium protein, unknown function
PF3D7_0522000 - Conserved Plasmodium protein, unknown function
PF3D7_0522100 - Conserved Plasmodium protein, unknown function
PF3D7_0522200 - Transcription initiation factor TFIID subunit 10, putative
PF3D7_0522300 - 18S rRNA (guanine-N(7))-methyltransferase, putative
PF3D7_0522400 - Conserved Plasmodium protein, unknown function
PF3D7_0522500 - 50S ribosomal protein L17, apicoplast, putative
PF3D7_0522600 - Magnesium transporter NIPA, putative
PF3D7_0522700 - Iron-sulfur cluster assembly protein SufA
PF3D7_0522800 - Pre-mRNA-splicing factor BUD31, putative
PF3D7_0522900 - Zinc finger protein, putative
PF3D7_0523000 - Multidrug resistance protein 1
PF3D7_0523100 - Mitochondrial-processing peptidase subunit alpha, putative
PF3D7_0523200 - Heptatricopeptide repeat-containing protein, putative

Supplementary Figure 5. Treatment and parasitological history of the patient infected by the strain 9097.

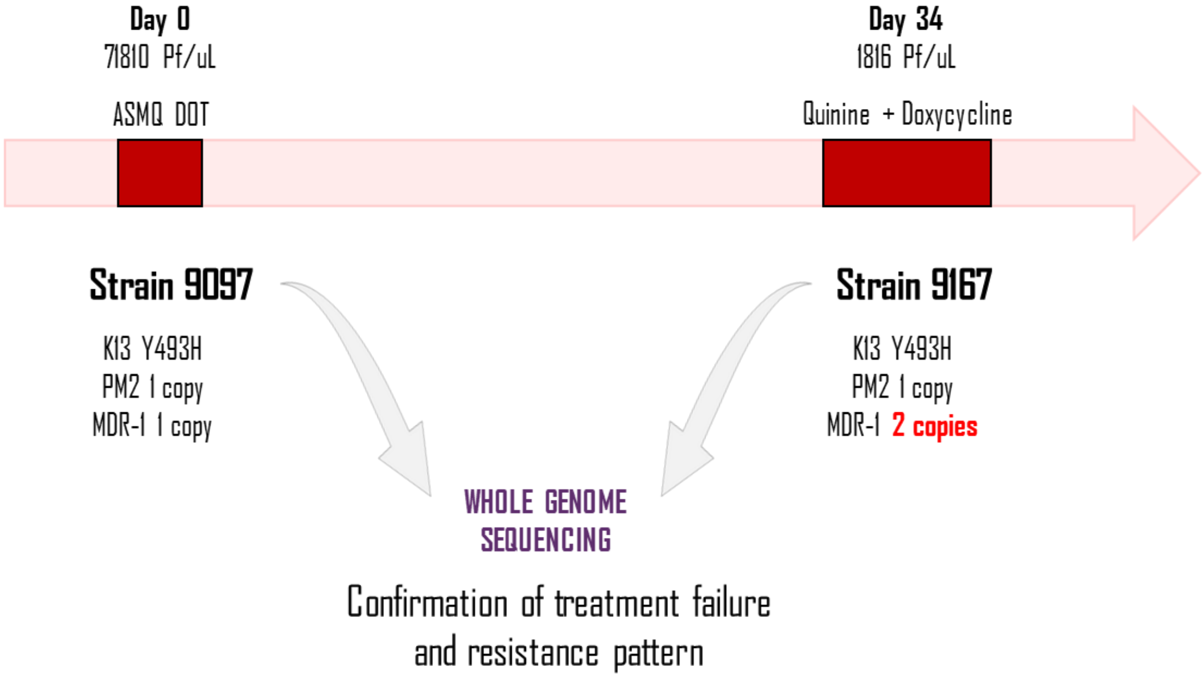

Supplementary Table 1. Evolution of *pfmdr1* and *pfpm2* copy numbers. P-values are based on two-sided tests.

Pfmdr1 – copy number value	Pfmdr1 Parental	Pfmdr1 Pressure 1	Pfmdr1 Pressure 2	Pfmdr1 Pressure 3	Pfmdr1 Pressure 4
**rep1**	1,050	1,174	1,517	1,895	1,790
**rep2**	1,176	1,138	1,710	1,833	2,025
**rep3**	1,061	1,289	1,543	1,816	1,915
**rep4**	0,979	1,028			2,219
**rep5**	0,976				2,101
**rep6**	1,078				2,022
**rep7**					1,918
**rep8**					1,787
**rep9**					1,890
**Mean**	**1,053**	**1,157**	**1,590**	**1,848**	**1,963**
**Std Dev**	0,074	0,108	0,105	0,042	0,143
**Dunnett's multiple comparisons test**	**Below threshold?**	**Summary**	**Adjusted P Value**		
**Pfmdr1 Parental vs. Pfmdr1 Pressure 1**	No	ns	0,4481		
**Pfmdr1 Parental vs. Pfmdr1 Pressure 2**	Yes	****	**5.16×10^{-6}**		
**Pfmdr1 Parental vs. Pfmdr1 Pressure 3**	Yes	****	**1.13×10^{-8}**		
**Pfmdr1 Parental vs. Pfmdr1 Pressure 4**	Yes	****	**6.00×10^{-12}**		
**Shapiro-Wilk test**	**Pfmdr1 Parental**	**Pfmdr1 Pressure 1**	**Pfmdr1 Pressure 2**	**Pfmdr1 Pressure 3**	**Pfmdr1 Pressure 4**
**P value**	0,4316	0,9563	0,2377	0,3932	0,6487
**Brown-Forsythe test - P value**	0,4665				
**Are SDs significantly different (P < 0.05)?**	No				

Pfpm2– copy number value	Pfpm2 Parental	Pfpm2 Pressure 1	Pfpm2 Pressure 2	Pfpm2 Pressure 3	Pfpm2 Pressure 4
**rep1**	1,260	1,253	0,859	0,846	0,912
**rep2**	1,338	0,958	1,138	0,768	1,021
**rep3**	1,199	1,058	1,120	0,996	1,060
**rep4**	0,761	1,118			0,949
**rep5**	0,853				0,973
**rep6**	0,869				0,930
**rep7**					0,870
**rep8**					0,842
**rep9**					0,818
**Mean**	**1,047**	**1,097**	**1,039**	**0,870**	**0,931**
**Std Dev**	0,247	0,123	0,156	0,116	0,080
**Dunnett's T3 multiple comparisons test**	**Below threshold?**	**Summary**	**Adjusted P Value**		
**Pfpm2 Parental vs. Pfpm2 Pressure 1**	No	ns	0,986221		
**Pfpm2 Parental vs. Pfpm2 Pressure 2**	No	ns	0,999994		
**Pfpm2 Parental vs. Pfpm2 Pressure 3**	No	ns	0,512959		
**Pfpm2 Parental vs. Pfpm2 Pressure 4**	No	ns	0,718289		
**Shapiro-Wilk test**	**Pfpm2 Parental**	**Pfpm2 Pressure 1**	**Pfpm2 Pressure 2**	**Pfpm2 Pressure 3**	**Pfpm2 Pressure 4**
**P value**	0,2244	0,9672	0,1101	0,6556	0,9405
**Brown-Forsythe test - P value**	0,0071				
**Are SDs significantly different (P < 0.05)?**	Yes				

Supplementary Table 2. List of amplified and deleted genes in Pressure 4 strain.

Gene_ID	*Parental*	*Pressure 4*	Differential	Gene description	Chromosome	Result
PF3D7_0522000	0,51	1,18	0,67	Uncharacterized protein	PF3D7_05	Amplified
PF3D7_0519500	1,06	1,76	0,7	poly(A)-specific ribonuclease	PF3D7_05	Amplified
PF3D7_0522300	0,69	1,45	0,76	18S rRNA (Guanine-N(7))-methyltransferase, putative	PF3D7_05	Amplified
PF3D7_0522200	0,78	1,55	0,77	Transcription initiation factor TFIID subunit 10, putative	PF3D7_05	Amplified
PF3D7_0522700	0,71	1,5	0,79	Iron-sulfur cluster assembly protein SufA	PF3D7_05	Amplified
PF3D7_0522500	0,68	1,48	0,8	50S ribosomal protein L17, apicoplast, putative	PF3D7_05	Amplified
PF3D7_0520600	0,91	1,72	0,81	Bis(5'-nucleosyl)-tetraphosphatase [asymmetrical]	PF3D7_05	Amplified
PF3D7_0520000	0,56	1,38	0,82	40S ribosomal protein S9, putative	PF3D7_05	Amplified
PF3D7_0523100	1,06	1,88	0,82	Alpha-MPP	PF3D7_05	Amplified
PF3D7_0522800	0,91	1,77	0,86	Pre-mRNA-splicing factor BUD31, putative	PF3D7_05	Amplified
PF3D7_0522900	1,19	2,09	0,9	Zinc finger protein, putative	PF3D7_05	Amplified
PF3D7_0521500	1,06	2,02	0,96	Ribosomal large subunit pseudouridylate synthase, putative	PF3D7_05	Amplified
PF3D7_0523200	1,03	2,02	0,99	Heptatricopeptide repeat-containing protein, putative	PF3D7_05	Amplified
PF3D7_0520700	1,15	2,14	0,99	CDC73 domain-containing protein, putative	PF3D7_05	Amplified
PF3D7_0519900	1,11	2,11	1	Uncharacterized protein	PF3D7_05	Amplified
PF3D7_0522100	1,07	2,07	1	RRM domain-containing protein	PF3D7_05	Amplified
PF3D7_0520100	0,92	1,92	1	Protein phosphatase PPM9, putative	PF3D7_05	Amplified
PF3D7_0521100	1,06	2,06	1	Uncharacterized protein	PF3D7_05	Amplified
PF3D7_0520500	0,92	1,93	1,01	Phosphomethylpyrimidine kinase, putative	PF3D7_05	Amplified
PF3D7_0521800	1,07	2,08	1,01	AFG1-like ATPase, putative	PF3D7_05	Amplified
PF3D7_0519700	0,82	1,83	1,01	FoP domain-containing protein, putative	PF3D7_05	Amplified
PF3D7_0522600	1,15	2,16	1,01	Inner membrane complex protein	PF3D7_05	Amplified
PF3D7_0520900	1,1	2,12	1,02	Adenosylhomocysteinase	PF3D7_05	Amplified
PF3D7_0521200	1,02	2,06	1,04	Uncharacterized protein	PF3D7_05	Amplified
PF3D7_0521300	1,07	2,12	1,05	Zinc finger protein, putative	PF3D7_05	Amplified
PF3D7_0519600	1,2	2,26	1,06	Zinc finger protein, putative	PF3D7_05	Amplified
PF3D7_0521900	1,16	2,22	1,06	Uncharacterized protein	PF3D7_05	Amplified
PF3D7_0522400	1,17	2,24	1,07	Chorein N-terminal domain-containing protein	PF3D7_05	Amplified
PF3D7_0519800	1,22	2,3	1,08	EELM2 domain-containing protein, putative	PF3D7_05	Amplified
PF3D7_0520400	1,13	2,21	1,08	Single-stranded DNA-binding protein, putative	PF3D7_05	Amplified
**PF3D7_0523000**	**1,17**	**2,25**	**1,08**	**Multidrug resistance protein 1**	**PF3D7_05**	**Amplified**
PF3D7_0520800	1,16	2,24	1,08	Uncharacterized protein	PF3D7_05	Amplified
PF3D7_0521700	1,11	2,23	1,12	ATP-dependent RNA helicase	PF3D7_05	Amplified
PF3D7_0520200	1,25	2,4	1,15	Mediator of RNA polymerase II transcription subunit 17, putative	PF3D7_05	Amplified
PF3D7_0521000	1,13	2,31	1,18	Uncharacterized protein	PF3D7_05	Amplified
PF3D7_0521400	1,1	2,42	1,32	SUN domain-containing protein	PF3D7_05	Amplified

PF3D7_0936000	0,57	0	0,57	Ring-exported protein 2	PF3D7_09	Deleted
PF3D7_0935500	0,67	0	0,67	Plasmodium RESA N-terminal domain-containing protein	PF3D7_09	Deleted
PF3D7_0936600	0,74	0	0,74	Plasmodium RESA N-terminal domain-containing protein	PF3D7_09	Deleted
PF3D7_0936200	0,75	0	0,75	Plasmodium RESA N-terminal domain-containing protein	PF3D7_09	Deleted
PF3D7_0935400	1,04	0,25	0,79	Gametocyte development protein 1	PF3D7_09	Deleted
PF3D7_0935700	0,86	0	0,86	Plasmodium RESA N-terminal domain-containing protein	PF3D7_09	Deleted
PF3D7_0935600	0,88	0	0,88	Gametocytogenesis-implicated protein	PF3D7_09	Deleted
PF3D7_0935900	0,88	0	0,88	Ring-exported protein 1	PF3D7_09	Deleted
PF3D7_0936400	0,89	0	0,89	Ring-exported protein 4	PF3D7_09	Deleted
PF3D7_0936800	0,91	0	0,91	Plasmodium RESA N-terminal domain-containing protein	PF3D7_09	Deleted
PF3D7_0935800	0,92	0	0,92	Cytoadherence linked asexual protein 9	PF3D7_09	Deleted
PF3D7_0936300	0,93	0	0,93	Ring-exported protein 3	PF3D7_09	Deleted
PF3D7_0936700	0,95	0	0,95	Lysophospholipase, putative	PF3D7_09	Deleted
PF3D7_0937000	0,97	0	0,97	Plasmodium RESA N-terminal domain-containing protein	PF3D7_09	Deleted
PF3D7_0937100	0,97	0	0,97	Uncharacterized protein	PF3D7_09	Deleted
PF3D7_0937200	1	0	1	Lysophospholipase, putative	PF3D7_09	Deleted
PF3D7_0936100	1,01	0	1,01	Early transcribed membrane protein	PF3D7_09	Deleted
PF3D7_0936500	1,05	0	1,05	Virulence-associated protein 1	PF3D7_09	Deleted

Supplementary Table 3. Characteristics and *in vitro* results of samples selected. Isolates noted with * have been used for chromosome 5 region analysis presented in Supplementary Figure 4. TES = Therapeutic Efficacy Study, ASAQ = Artesunate Amodiaquine, ASPI = Artesunate Pyronaridine, ASMQ = Artesunate Mefloquine, Q+DOX = Quinine + Doxycycline, na = not available.

Strain	Category	Year	Study name	Province	Day of collection	TES treatment	PfK13	PfPm2 copy number	Pfmdr1 copy number	CRT 88	CRT 93	CRT 97	CRT 145	CRT 343	CRT 353	IC_{50} MQ (nM) - [^{3}H]-hypoxanthine incorporation	PSA (%)	Survival IC_{50} MQ+PPQ coexposure (nM)
**7742**	KEL1/PLA1	2016	TES 2016	Mondulkiri	D0	ASAQ	C580Y	> 1,5	< 1,5	N	T	H	F	M	G	8,55	80,55	25,73
**8197**	KEL1/PLA1	2017	TES 2017	Mondulkiri	D0	ASPY	C580Y	> 1,5	< 1,5	N	T	H	I	M	G	24,37	66,37	23,94
**8286**	KEL1/PLA1	2017	TES 2017	Pursat	D0	ASMQ	C580Y	> 1,5	< 1,5	N	S	H	F	M	G	9,82	64,64	10,98
**8315**	KEL1/PLA1	2017	TES 2017	Rattanakiri	D0	ASPY	C580Y	> 1,5	< 1,5	K	T	H	F	M	G	24,87	42,85	30,74
**8546**	KEL1/PLA1	2018	TES 2018	Kampong Speu	D0	ASMQ	C580Y	> 1,5	< 1,5	N	T	Y	F	M	G	50,82	27,50	31,84
**9025**	KEL1/PLA1	2019	TES 2019	Kampong Speu	D0	ASMQ	C580Y	> 1,5	< 1,5	N	T	Y	F	M	G	40,65	57,40	14,88
**9028**	KEL1/PLA1	2019	TES 2019	Pursat	D0	ASMQ	C580Y	> 1,5	< 1,5	N	T	Y	F	M	G	30,55	46,16	17,97
**9034***	KEL1/PLA1	2019	TES 2019	Pursat	D0	ASMQ	C580Y	> 1,5	< 1,5	N	T	Y	F	M	G	34,8	64,18	33,59
**9074**	KEL1/PLA1	2019	TES 2019	Kampong Speu	D0	ASMQ	C580Y	> 1,5	< 1,5	N	T	Y	F	M	G	48,32	50,56	28,90
**8916**	MQ-R	2018	TES 2018	Rattanakiri	D0	ASMQ	R539T	< 1,5	> 1,5	N	T	H	F	M	G	107,49	0,01	63,70
**9042**	MQ-R	2019	TES 2019	Rattanakiri	D0	ASMQ	C580Y	< 1,5	> 1,5	N	T	H	F	M	G	100,40	0,39	34,72
**9061**	MQ-R	2019	TES 2019	Rattanakiri	D0	ASMQ	C580Y	< 1,5	> 1,5	N	T	H	F	M	G	85,96	0,00	47,20
**9062**	MQ-R	2019	TES 2019	Rattanakiri	D0	ASMQ	C580Y	< 1,5	> 1,5	N	T	H	F	M	G	72,57	0,00	47,42
**9065**	MQ-R	2019	TES 2019	Rattanakiri	D0	ASMQ	C580Y	< 1,5	> 1,5	N	T	H	F	M	G	105,15	0,00	28,25
**9067**	MQ-R	2019	TES 2019	Rattanakiri	D0	ASMQ	C580Y	< 1,5	> 1,5	N	T	H	F	M	G	69,56	0,00	63,21
**9069**	MQ-R	2019	TES 2019	Rattanakiri	D0	ASMQ	C580Y	< 1,5	> 1,5	N	T	H	F	M	G	71,20	0,00	29,06
**9071**	MQ-R	2019	TES 2019	Rattanakiri	D0	ASMQ	C580Y	< 1,5	> 1,5	N	T	H	F	M	G	93,69	0,00	52,52
**9077**	MQ-R	2019	TES 2019	Rattanakiri	D0	ASMQ	C580Y	< 1,5	> 1,5	N	T	H	F	M	G	125,58	0,00	37,62
**9078**	MQ-R	2019	TES 2019	Rattanakiri	D0	ASMQ	C580Y	< 1,5	> 1,5	N	T	H	F	M	G	72,81	0,00	26,38
**9080**	MQ-R	2019	TES 2019	Rattanakiri	D0	ASMQ	C580Y	< 1,5	> 1,5	N	T	H	F	M	G	77,26	0,00	63,69

**9081**	MQ-R	2019	TES 2019	Rattanakiri	D0	ASMQ	C580Y	< 1,5	> 1,5	N	T	H	F	M	G	89,05	0,18	57,64
**9093**	MQ-R	2019	TES 2019	Kampong Speu	D0	ASMQ	Y493H	< 1,5	> 1,5	N	T	H	F	M	G	118,95	1,41	67,58
**9099**	MQ-R	2019	TES 2019	Rattanakiri	D0	ASMQ	C580Y	< 1,5	> 1,5	N	T	H	F	M	G	147,89	0,00	54,92
**9128**	MQ-R	2019	TES 2019	Kampong Speu	D0	ASMQ	Y493H	< 1,5	> 1,5	N	T	H	F	M	G	126,47	0,00	83,84
**9167**	MQ-R	2019	TES 2019	Pursat	D34	Q+DOX	Y493H	< 1,5	> 1,5	N	T	H	F	M	G	90,36	0,00	46,51
**9175**	MQ-R	2019	TES 2019	Pursat	D40	Q+DOX	Y493H	< 1,5	> 1,5	N	T	H	F	M	G	83,61	0,70	67,23
**8944**	Sensitive	2018	TES 2018	Rattanakiri	D0	ASMQ	WT	< 1,5	< 1,5	N	T	H	F	M	G	20,77	0,00	23,70
**9032**	Sensitive	2019	TES 2019	Rattanakiri	D0	ASMQ	WT	< 1,5	< 1,5	N	T	H	F	M	G	51,95	0,00	23,60
**9037**	Sensitive	2019	TES 2019	Rattanakiri	D0	ASMQ	WT	< 1,5	< 1,5	N	T	H	F	M	G	54,68	0,00	20,26
**9046**	Sensitive	2019	TES 2019	Rattanakiri	D0	ASMQ	WT	< 1,5	< 1,5	N	T	H	F	M	G	35,39	0,00	21,11
**9049**	Sensitive	2019	TES 2019	Rattanakiri	D0	ASMQ	WT	< 1,5	< 1,5	N	T	H	F	M	G	52,15	0,00	27,32
**9097**	*Parental*	2019	TES 2019	Pursat	D0	ASMQ	Y493H	< 1,5	< 1,5	N	T	H	F	M	G	49,05	2,49	21,70
**9070***	PPQ-R SuppFig4	2019	TES 2019	Rattanakiri	D0	ASMQ	C580Y	< 1,5	< 1,5	K	T	H	F	M	G	41,97	39,61	na
**9083***	PPQ-R SuppFig4	2019	TES 2019	Kampong Speu	D0	ASMQ	C580Y	< 1,5	< 1,5	N	T	Y	F	M	G	50,71	56,37	na
**9088***	PPQ-R SuppFig4	2019	TES 2019	Kampong Speu	D0	ASMQ	C580Y	< 1,5	< 1,5	N	T	Y	F	M	G	42,06	25,61	na
