# Supplementary material for: Tolerance of Plasmodium falciparum mefloquine-resistant clinical isolates to mefloquine-piperaquine with implications for triple artemisinin-based combination therapies
Source: Nat Commun. 2025 Nov 27;16:10634. doi: 10.1038/s41467-025-65629-8 (PMC12660809; doi:10.1038/s41467-025-65629-8)
Supplement: Supplementary file 3 — Description of Additional Supplementary Files [file 41467_2025_65629_MOESM3_ESM.pdf]

Description of Additional Supplementary Files

File Name: Supplementary Data 1
Description: this dataset presents the primers sequences used in this manuscript
