# Supplementary material for: Tolerance of Plasmodium falciparum mefloquine-resistant clinical isolates to mefloquine-piperaquine with implications for triple artemisinin-based combination therapies
Source: Nat Commun. 2025 Nov 27;16:10634. doi: 10.1038/s41467-025-65629-8 (PMC12660809; doi:10.1038/s41467-025-65629-8)
Supplement: Supplementary file 5 — Reporting Summary [file 41467_2025_65629_MOESM5_ESM.pdf]

nature portfolio

Corresponding author(s): Benoit Witkowski
Last updated by author(s): Oct 14, 2025

Reporting Summary

Nature Portfolio wishes to improve the reproducibility of the work that we publish. This form provides structure for consistency and transparency in reporting. For further information on Nature Portfolio policies, see our Editorial Policies and the Editorial Policy Checklist.

Statistics

For all statistical analyses, confirm that the following items are present in the figure legend, table legend, main text, or Methods section.

n/a	Confirmed	
☐	☒	The exact sample size (*n*) for each experimental group/condition, given as a discrete number and unit of measurement
☐	☒	A statement on whether measurements were taken from distinct samples or whether the same sample was measured repeatedly
☐	☒	The statistical test(s) used AND whether they are one- or two-sided<br>*Only common tests should be described solely by name; describe more complex techniques in the Methods section.*
☒	☐	A description of all covariates tested
☐	☒	A description of any assumptions or corrections, such as tests of normality and adjustment for multiple comparisons
☐	☒	A full description of the statistical parameters including central tendency (e.g. means) or other basic estimates (e.g. regression coefficient) AND variation (e.g. standard deviation) or associated estimates of uncertainty (e.g. confidence intervals)
☐	☒	For null hypothesis testing, the test statistic (e.g. *F*, *t*, *r*) with confidence intervals, effect sizes, degrees of freedom and *P* value noted<br>*Give P values as exact values whenever suitable.*
☒	☐	For Bayesian analysis, information on the choice of priors and Markov chain Monte Carlo settings
☒	☐	For hierarchical and complex designs, identification of the appropriate level for tests and full reporting of outcomes
☒	☐	Estimates of effect sizes (e.g. Cohen's *d*, Pearson's *r*), indicating how they were calculated

Our web collection on statistics for biologists contains articles on many of the points above.

Software and code

Policy information about availability of computer code

Data collection	MicroBeta Workstation Software, PerkinElmer<br>CFX Maestro, version 2.3
Data analysis	MicroBeta Workstation Software, PerkinElmer<br>GraphPad Prism 7.0 and the ICEstimator software (http://www.antimalarial-icestimator.net)<br>All scripts used for data processing and analysis in this study are available on GitHub at https://github.com/Rcoppee/MQ-PPQ_pressure. A permanent archived version of the repository is available via Zenodo at https://doi.org/10.5281/zenodo.17283874.

For manuscripts utilizing custom algorithms or software that are central to the research but not yet described in published literature, software must be made available to editors and reviewers. We strongly encourage code deposition in a community repository (e.g. GitHub). See the Nature Portfolio guidelines for submitting code & software for further information.

Data

Policy information about availability of data

All manuscripts must include a data availability statement. This statement should provide the following information, where applicable:

- Accession codes, unique identifiers, or web links for publicly available datasets
- A description of any restrictions on data availability
- For clinical datasets or third party data, please ensure that the statement adheres to our policy

The source data underlying Figures 1a-d, 2a-c, 3, 4a-b, 5a-b, 6, Supplementary Figures 1 and 2 are provided as a Source Data file. Due to the large file size, the data underlying Supplementary Figure 3 are available from the corresponding authors upon reasonable request. The next-generation sequencing data generated in this study have been deposited in the European Nucleotide Archive (ENA) database under accession code PRJEB85790 (https://www.ebi.ac.uk/ena/browser/view/PRJEB85790)).

Research involving human participants, their data, or biological material

Policy information about studies with human participants or human data. See also policy information about sex, gender (identity/presentation), and sexual orientation and race, ethnicity and racism.

Reporting on sex and gender	Not applicable
Reporting on race, ethnicity, or other socially relevant groupings	Not applicable
Population characteristics	Cambodian patients infected with P. falciparum parasites, male and female, aged more than 2 years old with a parasitemia between 500-100000 parasites/ul. Pregnant woman were excluded.
Recruitment	This study is based on malaria parasites isolated from human subjects enrolled in therapeutic efficacy studies and further adapted to continuous in vitro culture. No clinical data are exploited within this study. Recruitment was performed during passive cases detection in Cambodia. The study being based on parasites biology after in vitro culture adaptation, no selection biais have been identified.
Ethics oversight	Cambodian National Ethics Committee for Health Research (identifiers: NECHR #086 (Therapeutic Efficacy Study 2017), NECHR #087 (Therapeutic Efficacy Study 2017), NECHR #092 (Therapeutic Efficacy Study 2019), NECHR #099 (Therapeutic Efficacy Study 2016), NECHR #136 (Therapeutic Efficacy Study 2016) and NECHR #106 (Therapeutic Efficacy Study 2018)) and WHO Western Pacific Regional Office (WPRO) Ethical Review Committee.

Note that full information on the approval of the study protocol must also be provided in the manuscript.

Field-specific reporting

Please select the one below that is the best fit for your research. If you are not sure, read the appropriate sections before making your selection.

☒ Life sciences ☐ Behavioural & social sciences ☐ Ecological, evolutionary & environmental sciences

For a reference copy of the document with all sections, see nature.com/documents/nr-reporting-summary-flat.pdf

Life sciences study design

All studies must disclose on these points even when the disclosure is negative.

Sample size	No formal sample size calculation was performed. Sample sizes were determined empirically based on previous experiments with culture-adapted isolates and the availability of field-collected samples. They were sufficient to carry out all required experiments with adequate statistical power and consistent with standards commonly used in the field. The number of biological replicates included allowed detection of reproducible and biologically meaningful differences across groups, ensuring robust and reliable conclusions.
Data exclusions	No data was excluded.
Replication	All experiments were performed with at least three independent replicates. Biological replicates, including multiple strains, were used to assess in vitro drug efficacy within each parasite group of field isolates. The drug pressure experiment was conducted using a single culture, but phenotypic changes were evaluated using the same replication and assessment approaches as for the other experiments.
Randomization	Randomization is not relevant to the study design mainly based on in vitro data
Blinding	Blinding was not applied during data collection or analysis. However, most experiments (gene copy number analysis, sequencing, and radioactivity incorporation assays) relied on objective quantitative measurements, for which investigator bias was unlikely to influence the results. Regarding microscopy, potential reading bias was minimized by ensuring sufficient event counts, and all slides were examined by an experienced and qualified microscopist.

Reporting for specific materials, systems and methods

We require information from authors about some types of materials, experimental systems and methods used in many studies. Here, indicate whether each material, system or method listed is relevant to your study. If you are not sure if a list item applies to your research, read the appropriate section before selecting a response.

Materials & experimental systems

n/a	Involved in the study
☒	☐ Antibodies
☒	☐ Eukaryotic cell lines
☒	☐ Palaeontology and archaeology
☒	☐ Animals and other organisms
☒	☐ Clinical data
☒	☐ Dual use research of concern
☒	☐ Plants

Methods

n/a	Involved in the study
☒	☐ ChIP-seq
☒	☐ Flow cytometry
☒	☐ MRI-based neuroimaging

Plants

Seed stocks	Not applicable
Novel plant genotypes	Not applicable
Authentication	Not applicable
